# Anti-idiotypic antibodies reduce efficacy of the attenuated vaccine against highly pathogenic PRRSV challenge

**DOI:** 10.1186/1746-6148-10-39

**Published:** 2014-02-08

**Authors:** Ying Yu, Xuehui Cai, Gang Wang, Ning Kong, Yonggang Liu, Yihong Xiao, Chong Zhang, Yang Mu, Shuqi Xiao, Qin Zhao, Chengbao Wang, Gaiping Zhang, Julian A Hiscox, En-Min Zhou

**Affiliations:** 1 Department of Preventive Veterinary Medicine, College of Veterinary Medicine, Northwest A&F University, Yangling, Shaanxi 712100, China; 2 State Key Laboratory of Veterinary Biotechnology, Harbin Veterinary Research Institute of Chinese Academy of Agriculture Science, Harbin 150001, China; 3 Department of Preventive Veterinary Medicine, College of Veterinary Medicine, Shandong Agricultural University, Taian, Shandong 271018, China; 4 College of Animal Science and Veterinary Medicine, Henan Agricultural University, Zhengzhou, Henan 450002, China; 5 Department of Infection Biology, Institute of Infection and Global Health, University of Liverpool, Liverpool L3 5RF, UK

**Keywords:** Anti-idiotype, Cytokines, PRRSV, Vaccination

## Abstract

**Background:**

The inability of current vaccines to provide effective protection against porcine reproductive and respiratory syndrome virus (PRRSV) infection is not fully understood. One of the reasons might be the presence of anti-idiotypic antibodies (Ab2s) to the envelope glycoprotein GP5 induced by PRRSV infection since our previous studies demonstrated the presence of auto-Ab2s (aAb2s) in pigs infected with PRRSV. To test this hypothesis, PRRSV negative piglets were injected with a monoclonal Ab2 (Mab2-5G2) and aAb2s that are specific for anti-GP5 antibody, vaccinated with the attenuated PRRSV vaccine CH-1R and then challenged with the highly pathogenic PRRSV HuN4 strain. The animals were evaluated for clinical signs, pathological changes of the thymus and lungs, viremia, levels of serum antibodies and cytokines.

**Results:**

The piglets injected with Mab2-5G2 or aAb2, and who received the attenuated PRRSV vaccine CH-1R before challenge, produced high levels of anti-N antibodies, IL-2 and IL-4, but low levels of neutralizing antibodies. After PRRSV HuN4 challenge, the animals showed obvious clinical signs, including lung lesions, severe thymus atrophy and decreased production of IL-4 and higher level of viremia.

**Conclusion:**

When anti-GP5 Ab2s are present, the use of attenuated PRRSV vaccine CH-1R against HP-PRRSV infection is not recommended. It can result in poor health status with pneumonia and thymus atrophy.

## Background

Porcine reproductive and respiratory syndrome (PRRS) has emerged as one of the most important swine diseases worldwide since its appearance in the late 1980s. PRRS virus (PRRSV), the causative agent of PRRS, is an enveloped RNA virus belonging to the family *Arteriviridae*. The virus genome, like other members of the family [1], contains nine open reading frames [2-4]. PRRSV infects macrophages, damages the immune system and causes severe host immune response disorders. This results in prolonged viremia, transiently diminishing T-cell immunity [5-7] and delayed or low neutralizing antibody response against the envelope glycoprotein 5 (GP5) and the matrix (M) protein [8,9].

The immune network theory proposed by Jerne [10] suggests that idiotype (Id) and anti-Id interactions control the immune response to an antigen via either positive (enhancing) or negative (suppressing) feedback mechanisms [11-14]. Our previous studies demonstrated that PRRSV infection induced the production of auto-anti-Id (aAb2s) specific for antibodies to the GP5 and M proteins [15,16]. The pigs produced aAb2s during the early stage of infection (35 days post infection) cleared the virus, whereas those pigs who had aAb2s at the later stage of infection (77 days post infection) became virus carriers. These results suggested the presence of aAb2s specific for anti-GP5 or anti-M antibodies may interfere with the immune responses against PRRSV infection.

At present, both live attenuated and inactivated PRRSV vaccines cannot provide sustainable disease control [17]. The efficacy of PRRSV vaccination against PRRSV infection may be related to the cellular immune responses induced by vaccination [18-20]. In addition, the live attenuated vaccines have protective value against PRRS clinical disorders, but cannot prevent the disease or shorten persistent infection in individual pigs, resulting in long-term circulation of virus within swine herds [21] and the potential to revert to virulence [22].

The inability of current vaccines to provide effective protection against PRRSV infection is not fully understood. We hypothesize one of the reasons for this is that PRRSV infection induces the production of anti-Ids to anti-GP5 and when these anti-Ids are present, they interfere with the efficacy of the attenuated PRRSV vaccine (CH-1R) to protect against highly pathogenic (HP) PRRSV infection. To test this hypothesis, piglets free of PRRSV were injected with either a monoclonal Ab2 (designated Mab2-5G2) [23] or aAb2s specific to anti-GP5 antibody. Piglets then received CH-1R and were challenged with the HP-PRRSV HuN4 strain. The results showed that piglets who received Mab2-5G2 or aAb2s and were vaccinated with CH-1R before HP-PRRSV infection produced high levels of serum IL-2 and IL-4. After infection, these animals showed obvious clinical signs of wheezing, anhelation, severe lethargy, anorexia, purulent secretion and emaciation. Additionally there were signs of depletion of cortical thymocytes or severe thymus atrophy with decreased production of IL-4, low levels of neutralizing antibodies and a high level of viremia at the early stage of infection. These results suggested that Ab2s specific for anti-PRRSV GP5 antibody interfere with the immune responses against the attenuated PRRSV vaccine CH-1R. Further study is needed to identify, at the molecular and cellular levels, the roles of the idiotype network in PRRSV immunity.

## Results

### Clinical presentation

No piglets showed clinical signs before HP-PRRSV challenge, consistent with their disease free status. After the challenge with HP-PRRSV, Group 1, 2 and 5 piglets showed wheezing and anhelation from 3 to 14 DPC with the average scores of 1.43 to 3.67 (Table 1). Severe lethargy, anorexia and emaciation were observed from 3 to 16 DPC and purulent secretion from the nose between 7 to 21 DPC. One piglet from Group 1 and one piglet from Group 5 died naturally from PRRS at 16 DPC and 18 DPC, respectively. Group 3 and 4 piglets showed transient anhelation from 7 to 11 DPC with average scores of 0.5 to 1.12 (Table 1) without other obvious clinical signs and piglets in Group 6 had no clinical signs.

**Table 1:** Respiratory scores measured on different DPCs

**DPCs**	**Scores from six groups of piglets**^**1**^					
	**1**	**2**	**3**	**4**	**5**	**6**
0	0 (6)	0 (6)	0 (6)	0 (6)	0 (6)	0 (6)
3	1.43 ± 0.20 (6)^a^	1.67 ± 0.21 (6)^a^	0 (6)^b^	0 (6)^b^	1.60 ± 0.24 (6)^a^	0 (6)
7	2.71 ± 0.29 (6)^a^	3.0 ± 0.37 (6)^a^	0.71 ± 0.29 (6)^b^	1.12 ± 0.31 (6)^b^	3.0 ± 0.32 (6)^a^	0 (6)
10	3.25 ± 0.48 (6)^a^	3.5 ± 0.22 (6)^a^	0.50 ± 0.29 (6)^b^	0.83 ± 0.31 (6)^b^	3.33 ± 0.33 (6)^a^	0 (6)
14	3.5 ± 0.29 (6)^a^	2.33 ± 0.21 (6)^a^	0 (6)^b^	0.33 ± 0.22 (6)^b^	3.67 ± 0.33 (6)^a^	0 (6)
21	1.33 ± 0.33 (2)^2^	1.67 ± 0.33 (3)	0 (3)	0 (3)	1.50 ± 0.50 (2)^2^	0 (6)
28	0.67 ± 0.33 (2)	1.33 ± 0.33 (3)	0 (3)	0 (3)	0.5 ± 0.5 (2)	0 (6)

^1^Respiratory scores were given from 0 to 4: 0, normal; 1, tachypnea when stressed; 2, tachypnea at rest; 3, tachypnea and dyspnea at rest; 4, severe tachypnea and dyspnea with labored, jerky breathing. Each number represents the mean scores (±S.D.) generated from six groups of piglets on different DPCs and analyzed with ANOVA.

^2^The numbers in parentheses were the numbers of piglets measured on different DPCs and one piglet from each of Group 1 and Group 5 died from PRRS on 16 DPC and 18 DPC, respectively.

^a,b^Values with different superscripts were significantly different (*P* < 0.05).

**Figure 1:**
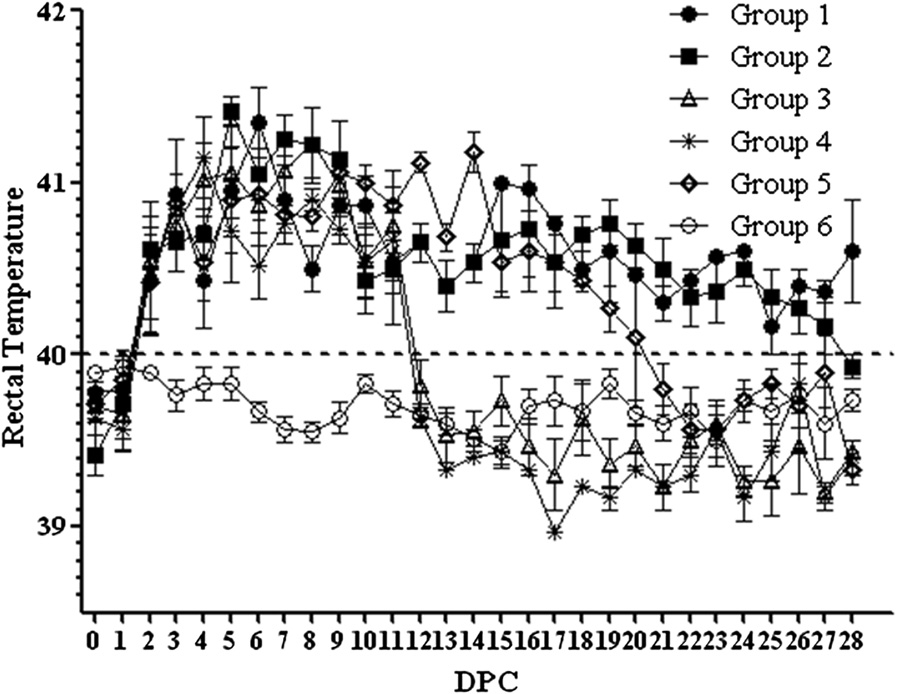
**Rectal temperature (°C) changes in piglets after HP-PRRSV infection.** Group 1 to 4 piglets were given Mab2-5G2, aAb2s, 7H12 and normal swine IgG on 0 DPI, respectively, vaccinated on 14 and 28 DPI and challenged with HP-PRRSV on 0 DPC. Group 5 piglets were challenged with HP-PRRSV only and Group 6 piglets received PBS. Each point represents the mean (±S.D) generated from all piglets in each group on each DPC.

The average rectal temperature of Group 1 and 2 piglets was similar, with the highest temperature of 41.5°C between 5 and 6 DPC (Figure 1). The temperature remained above 40.0°C to the end of the experiment. The rectal temperature of Group 3 and 4 piglets started to rise from 2 DPC and returned to normal from 12 DPC to the end of the experiment. The rectal temperature of Group 5 piglets remained above 40°C from 2 to 20 DPC and dropped below 40.0°C from 21 DPC to the end of the experiment. Group 6 piglets had a normal rectal temperature.

### Visual and histological observations

**Figure 2:**
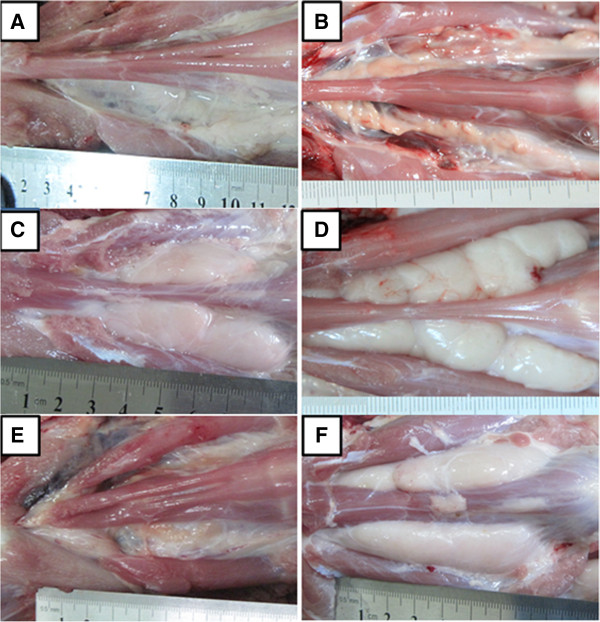
**Gross pathology of thymus on 28 DPC.** Thymus of a piglet from Group 1 **(A)**, Group 2 **(B)** and Group 5 **(E)** showed serious atrophy. Thymus of a piglet from Group 3 **(C)** and Group 4 **(D)** showed slight atrophy and thymus of an age-matched control piglet from Group 6 **(F)** was normal.

Severe thymus atrophy was observed for Group 1, 2 and 5 piglets on 14 DPC (data not shown) and 28 DPC (Figure 2A, 2B, 2E). One piglet from Group 1 and one from Group 5 died naturally from PRRS on 16 DPC and 18 DPC, respectively, accompanied with severe thymus atrophy (data not shown). The thymus atrophy of piglets infected with HP-PRRSV was similar to that observed in the previously reported study [24]. Thymus atrophy of Group 3 (Figure 2C) and Group 4 (Figure 2D) piglets was weaker than that of Group 1 and 2 piglets on 28 DPC, and the thymuses of Group 6 piglets were normal (Figure 2F).

**Figure 3:**
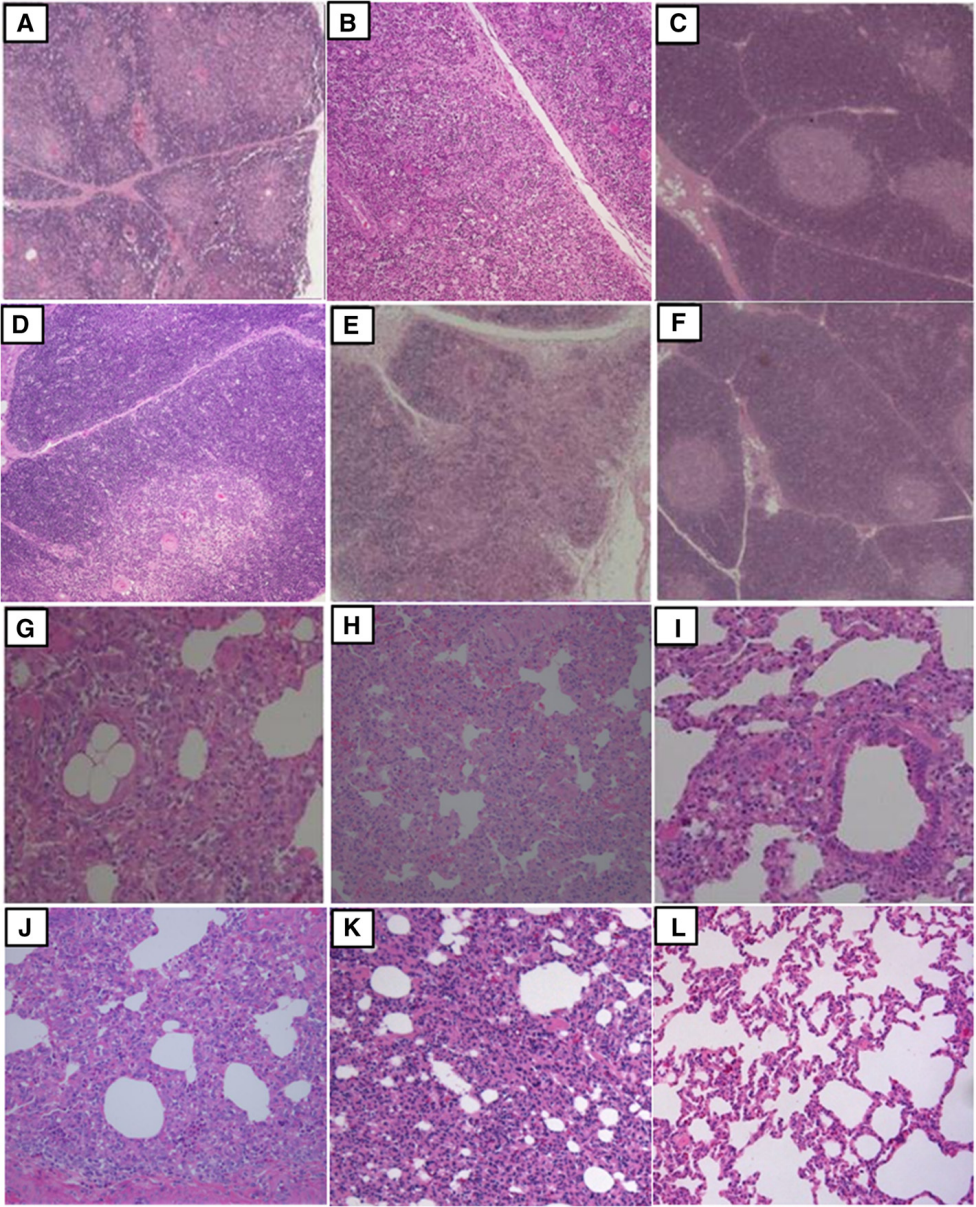
**Pathological examinations of the thymus and lung on 28 DPC.** Thymus of a piglet from Group 1 **(A)**, Group 2 **(B)**, and Group 5 **(E)** showed blurred boundaries between the thymus cortex and medulla. Thymus of a piglet from Group 3 **(C)**, Group 4 **(D)** and Group 6 **(F)** showed no microscopic lesions (magnification × 50). Lungs of a piglet from Group 1 **(G)**, Group 2 **(H)** and Group 5 **(K)** showed interstitial pneumonia lesions, lungs of a piglet from Group 3 **(I)** and Group 4 **(J)** showed mild interstitial pneumonia, and a Group 6 piglet **(L)** was normal (magnification × 200).

Thymuses collected on 28 DPC from Group 1 piglets showed typical depletion of cortical thymocytes accompanied by blurred boundaries between the thymus cortex and medulla as indicated by histology (Figure 3A). Similar microscopic lesions were seen in the thymuses of Group 2 and 5 piglets (Figure 3B, 3E). Group 3, 4 and 6 piglets showed no microscopic lesions of the thymus (Figure 3C, 3D, 3F). Interstitial pneumonia lesions were observed in Group 1, 2 and 5 piglets on 28 DPC (Figure 3G, 3H, 3K). The lung tissues from Group 3 and 4 piglets were widened slightly on 28 DPC (Figure 3I, 3J), and that from Group 6 piglets were normal (Figure 3L).

### Viremia

Viremia of piglets from Groups 1–5 was detected starting on 3 DPC (Table 2). The amount of virus was significantly higher in Group 1 and 2 piglets who received Mab2-5G2 and aAb2s, respectively, than that in piglets from other Groups. The highest amount of virus present in the sera of Group 1 and 2 piglets was on 7 DPC with a mean titer of 10^8.0^ copies/ml and 10^8.4^ copies/ml, respectively, and this started to decline from 10 DPC but remained detectable at 21 DPC. Viremia in Group 3 and 4 piglets peaked on 7 DPC with a mean titer of 10^6.2^ copies/ml and 10^6.0^ copies/ml, respectively, which were significantly lower (*P* < 0.05) than that from Group 1 and 2 piglets, and became undetectable on 21 DPC. The amount of virus in Group 5 piglets on 3 DPC was significantly lower than that from Group 1and 2 piglets and the highest amount of virus was detected at 10 DPC with the mean titer of 10^8.2^ copies/ml, which dropped to 10^7.2^ copies/ml on 14 DPC. These values were significantly higher (*P* < 0.05) than those from Group 3 and 4 piglets and remained detectable on 21 DPC similar to values from Group 1 and 2 piglets (Table 2). No virus was detected in Group 6 piglets.

**Table 2:** Virus quantity in serum samples detected on different DPCs

**DPCs**	**Virus quantity from six groups of piglets**^**1**^					
	**1**	**2**	**3**	**4**	**5**	**6**
0	0 (6)	0 (6)	0 (6)	0 (6)	0 (6)	0 (6)
3	7.3 ± 0.3 (6)^a^	7.9 ± 0.6 (6)^a^	5.5 ± 0.3 (6)^b^	5.3 ± 0.2 (6)^b^	5.8 ± 0.2 (6)^b^	0 (6)
7	8.0 ± 0.2 (6) ^a^	8.4 ± 0.4 (6) ^a^	6.2 ± 0.3 (6) ^b^	6.0 ± 0.4 (6) ^b^	7.5 ± 0.3 (6) ^a^	0 (6)
10	7.1 ± 0.2 (6)^a^	7.5 ± 0.3 (6)^a^	6.0 ± 0.4 (6)^b^	5.8 ± 0.4 (6)^b^	8.2 ± 0.2 (6)^a^	0 (6)
14	6.0 ± 0.6 (6)^a^	6.4 ± 0.5 (6)^a^	4.8 ± 0.5 (6)^b^	5.0 ± 0.5 (6)^b^	7.2 ± 0.4 (6)^a^	0 (6)
21	2.7 ± 1.3 (2)^2^	3.0 ± 0.7 (2)	0 (3)	0 (3)	2.3 ± 2.3 (2)^2^	0 (3)

^1^Each number represents the mean virus copies/ml (log_10_) (±S.D.) generated from six groups of piglets on different DPCs and analyzed with ANOVA.

^2^Same as that in Table 1.

^a, b^Values with different superscripts were significantly different (*P* < 0.05).

### Serum concentrations of cytokines

**Figure 4:**
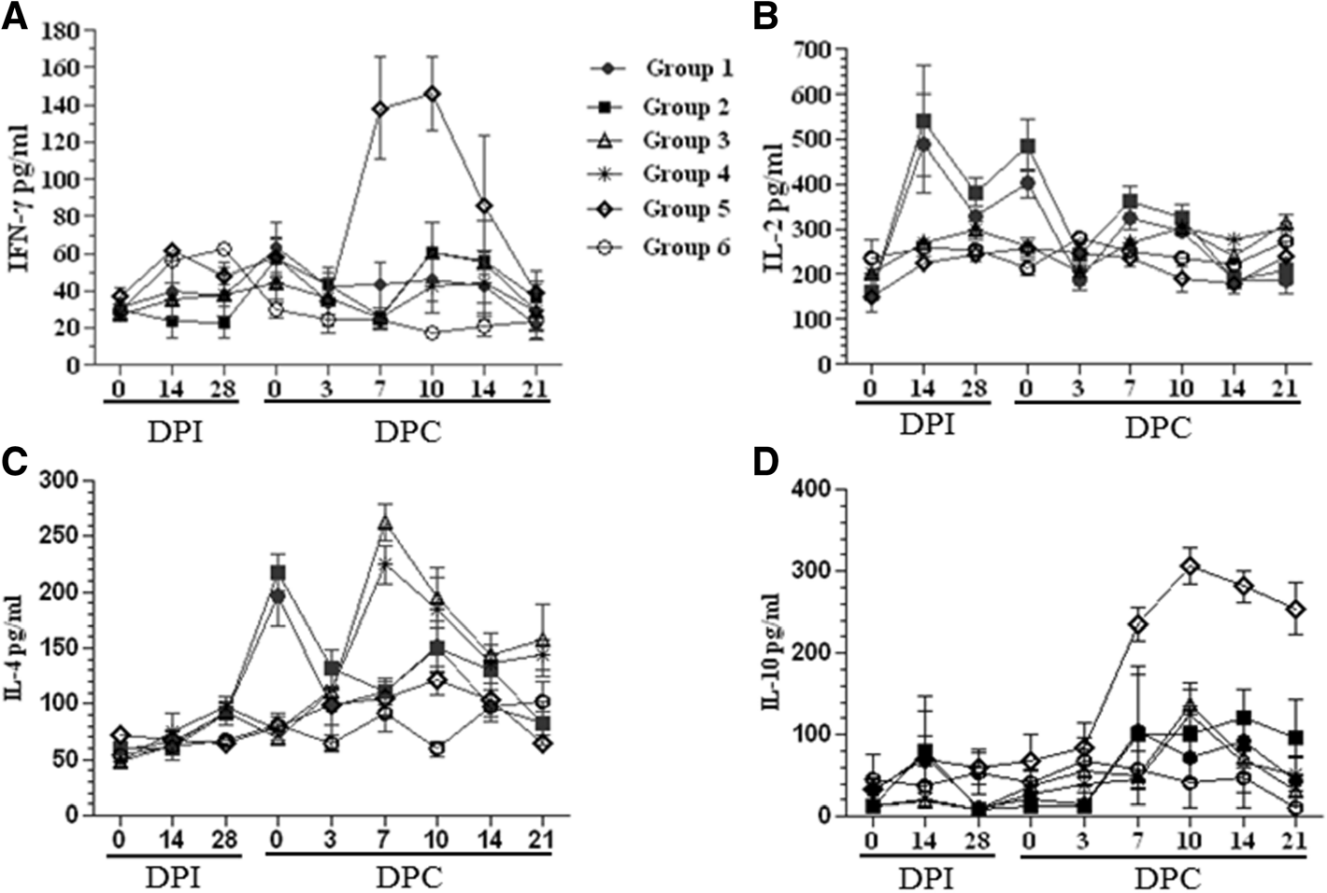
**Serum levels of cytokines.** Serum concentrations of IFN-γ **(A)**, IL-2 **(B)**, IL-4 **(C)** and IL-10 **(D)** were measured using the commercial ELISA kits (Market Inc, USA). Each point represents the mean value (±S.D) generated from six groups of piglets on different DPIs or DPCs.

The concentrations of IFN-γ, IL-2, IL-4 and IL-10 were determined for all serum samples (Figure 4). The serum concentrations of IFN-γ for Group 1–4 and Group 6 piglets were about 45 pg/ml in average for all serum samples, whereas the levels of IFN-γ from Group 5 piglets started to rise on 3 DPC and increased significantly (*P* < 0.05) on 10 DPC (131 pg/ml) (Figure 4A).

The mean serum IL-2 concentration from piglets in Group 1 and 2 was about 540 pg/ml and 460 pg/ml on 14 DPI and 0 DPC (42DPI), respectively. These were significantly higher (*P* < 0.05) than those obtained from the other four Groups of piglets with an average of 245 pg/ml for all samples (Figure 4B).

The level of IL-4 in sera from Group 1 and 2 piglets peaked on 35 DPI or 0 DPC with the average of 200 pg/ml and then declined to the base levels on 3 DPC (Figure 4C). In contrast, the level of IL-4 in serum from Group 3 and 4 piglets started to rise on 3 DPC, peaked on 7 DPC with an average of 220 pg/ml and then declined gradually. The IL-4 concentrations from Group 5 piglets were less than 110 pg/ml for all serum samples.

For the levels of serum IL-10, Group 5 piglets showed the increased levels of approximately 220 pg/ml on 7DPC, peaked with 374 pg/ml on 10 DPC and then declined gradually, but remained more than 200 pg/ml to 21 DPC (Figure 4D). The IL-10 concentrations from all serum samples of Group 1–4 and Group 6 piglets were less than 130 pg/ml (Figure 4D).

### Serum anti-PRRSV antibodies

**Figure 5:**
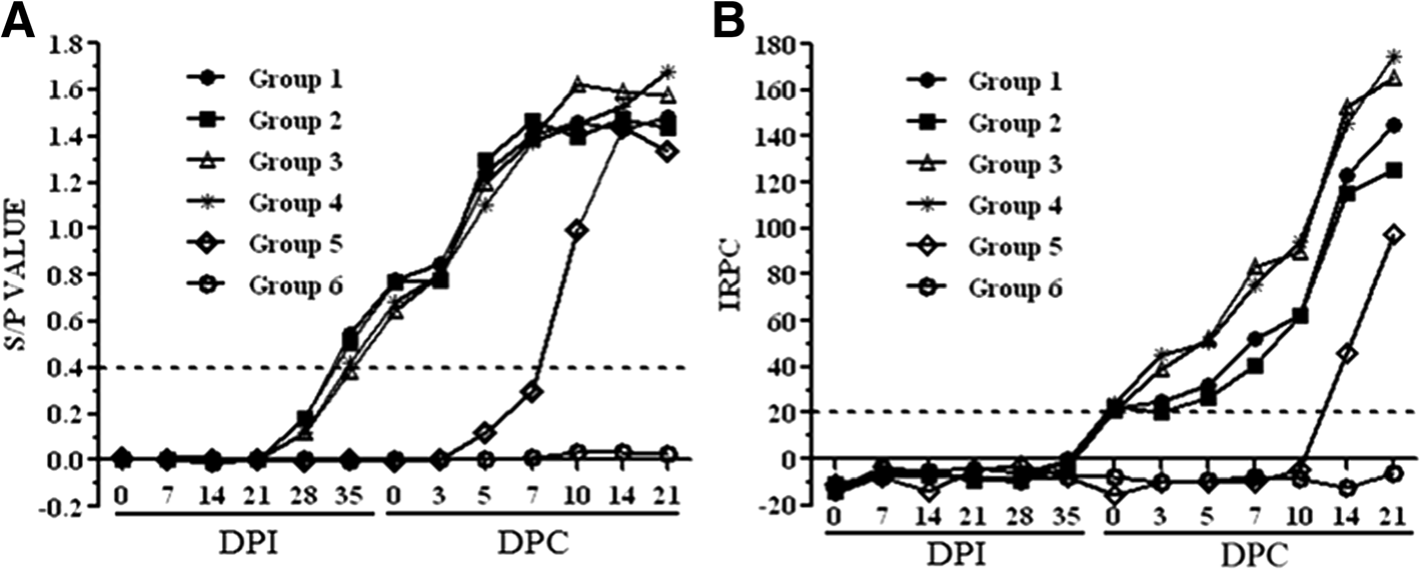
**Levels of serum antibodies.** Anti-N antibodies were detected using the HerdCheck® PRRS ELISA kit with the cutoff value of S/P ratio of 0.4 **(A)** and anti-GP2/GP5 antibodies were detected using the LIVTESTSUISPRRS A/S® kit with the relative index (expressed as a percentage) greater than 20 as positive **(B)**. Each point represents the mean (±S.D.) generated from six groups of piglets on different DPIs or DPCs.

Anti-PRRSV antibodies from all piglets were detected in sequential serum samples collected from 0 DPI to 21 DPC. The results of anti-N antibodies measured by IDEXX ELISA were shown in Figure 5A. Piglets in Groups 1 to 4 seroconverted as early as 35 DPI (21 days post vaccination) with an average S/P value of about 0.5 and rose quickly after HP-PRRSV challenge. In contrast, piglets in Group 5 seroconverted on 10 DPC with the average S/P value of 1.0 and peaked on 14 DPC (S/P = 1.42). Group 6 piglets had no detectable anti-N antibodies.

As shown in Figure 5B, serum anti-GP2/GP5 antibodies from Group 1 and 2 piglets were detected on 0 DPC with the IRPC value of ≥20 and gradually increased to a peak on 21 DPC with the IRPC value of 130. Similarly, piglets from Groups 3 and 4 also seroconverted on 0 DPC and gradually increased with the higher IRPC values on each DPC than that from Group 1 and 2 piglets and reached a peak on 21 DPC with the IRPC value of 170. In contrast, Group 5 piglets seroconverted on 14 DPC with the IRPC value of 46 and peaked on 21 DPC (IRPC = 97.51). Serum anti-GP2/GP5 antibodies in Group 6 piglets were negative.

### Neutralizing antibodies

Serum neutralizing antibodies against HP-PRRSV HuN4 strain were detected using a VN assay. As shown in Table 3, the neutralizing antibodies were detected in Group 1 and 2 piglets starting at 21 DPI with an average titer of 2.5 and peaked by 21 DPC. Group 3 and 4 piglets did not produce detectable neutralizing antibodies until 35 DPIs, and gradually increased to the peak on 21 DPC. Neutralizing antibodies from Group 5 piglets were detected from 14 DPC.

**Table 3:** Neutralizing antibodies detected from sera collected on different DPIs and DPCs

**DPIs/DPCs**	**Titers of neutralizing antibodies from six groups of piglets**^**1**^					
	**1**	**2**	**3**	**4**	**5**	**6**
DPIs						
0	0 (6) ^2^	0 (6)	0 (6)	0 (6)	0 (6)	0 (6)
7	0 (6)	0 (6)	0 (6)	0 (6)	0 (6)	0 (6)
14	0 (6)	0 (6)	0 (6)	0 (6)	0 (6)	0 (6)
21	2.3 (6)	2.7 (6)	0 (6)	0 (6)	0 (6)	0 (6)
28	3.3 (6)	3.3 (6)	0(6)	0 (6)	0 (6)	0 (6)
35	3.3 (6)	3.7 (6)	4.7 (6)	5.3 (6)	0 (6)	0 (6)
DPCs						
0	3.7 (6)	3.7 (6)	6 (6)	6.3 (6)	0 (6)	0 (6)
3	2.7 (6)	3.3 (6)	5.3 (6)	5.3 (6)	0 (6)	0 (6)
7	3.3 (6)	3.3 (6)	5.3 (6)	6 (6)	0 (6)	0 (6)
10	3.3 (6)	3.3 (6)	6.7 (6)	8 (6)	0 (6)	0 (6)
14	3.7 (6)	4.7 (6)	8.7 (6)	10.1(6)	5.3 (6)	0 (6)
21	6 (2)	6.7 (3)	10.7 (3)	13.3 (3)	12 (2)	0 (3)

^1^Neutralizing antibodies were detected to evaluate the ability of antibodies against Mab2-5G2 and aAb2s to neutralize PRRSV infection of Marc-145 cells. Briefly, a 2-fold diluted serum sample and a titer of 100 TCID_50_/mL was incubated for 1 h at 37°C and was transferred to a 96-well plate containing a Marc-145 cell monolayer. Neutralizing antibody titers were defined as the mean of reciprocal of the highest dilution of sera that inhibited 50% CPE.

^2^Each number represents the mean dilution of neutralizing antibody titers generated from six groups of piglets on different DPIs/DPCs. The numbers in parentheses were the numbers of piglets measured on different DPIs/DPCs.

## Discussion

This report describes that the presence of monoclonal anti-Id (Mab2-5G2) [23] or aAb2s [16] specific for anti-GP5 antibody interfered with the PRRSV attenuated vaccine, CH-1R, against HP-PRRSV infection. The ability of CH-1R to provide effective protection against a HP-PRRSV infection has been demonstrated in our previous study [24], in which, CH-1R immunization decreased and increased the serum level of IL-10 and IL-4, respectively, along with the increased animal’s health status. The results indicated that administration of Mab2-5G2 or aAb2s, to reproduce what would occur in a pig if it had been infected with PRRSV before vaccination, markedly reduced the ability of CH-1R in providing subsequent protection against HP-PRRSV infection. The selected amount of Mab2-5G2 or aAb2s was based on the measurement of aAb2s from pig sera (data not shown) which represented the amounts that would be generated if they were naturally infected with PRRSV.

HP-PRRSV infection alone induced severe thymus atrophy as demonstrated in previous findings [24,25]. In this study, piglets which received Mab2-5G2 or aAb2s and vaccination, also showed severe thymus atrophy after HP-PRRSV challenge (Figure 2). These findings indicated that presence of Mab2-5G2 or aAb2s prevents the efficacy of the CH-1R against HP-PRRSV-induced thymus atrophy attributable to the thymocytes apoptosis [25]. PRRSV infection induces cell apoptosis and this is related to the activity of the GP5 protein [26]. Since Mab2-5G2 and aAb2s were specific for anti-GP5 antibodies, they may act in concert with GP5 protein to induce apoptosis in thymocytes.

Swine possess the full repertoire of innate and adaptive immune responses to viral pathogens, but the response to PRRSV deviates from this model [27]. In general, Th1 responses are associated with IFN-γ and IL-2 production, which are correlated with the induction of cell-mediated immunity. PRRSV infection alone can induce high levels of IFN-γ as shown in this (Figure 4A) and previous studies [24,28,29]. In the current study, piglets which received the vaccination did not produce high levels of IFN-γ after HP-PRRSV challenge (Groups 3 and 4 in Figure 4A), indicating that the CH-1R down-regulated IFN-γ expression which was not affected by Mab2-5G2 or aAb2s (Group 1 and 2 in Figure 4A). The down-regulated IFN-γ expression by CH-1R and the high level of expression induced by HP-PRRSV infection showed that the IFN-γ mediated cell immune response were not capable of eliminating HP-PRRSV. At present, the role of IFN-γ in the immune response to CH-1R and HP-PRRSV infection is not clear and warrants further investigation.

Administration of Mab2-5G2 or aAb2s induced high concentrations of serum IL-2 on 14 DPI and 0 DPC before HP-PRRSV challenge (Figure 4B). However, piglets challenged with HP-PRRSV alone or who received CH-1R did not produce IL-2, which is consistent with other studies of PRRSV infection [19,30]. IL-2 is related to the Th1 responses and used to evaluate candidate vaccines to PRRSV and other viruses [17,31,32]. IL-2 is also necessary during T cell development in the thymus for the maturation of regulatory T cells (T-regs). After exiting from the thymus, T-regs prevent other T cells from recognizing and reacting against “self antigens” [33,34]. At present, the role of IL-2 in PRRSV infection was unknown. The poor health status of piglets in Group 1 and 2 who received Mab2-5G2 or aAb2s showed a high level of IL-2 before and after HP-PRRSV challenge (Figure 4B) and may have a negative effect on the immune responses against HP-PRRSV infection.

IL-4 is a key regulator in humoral and adaptive immunity in association with the down-regulation of Th1 cell responses and promotion of Th2 cell development [35]. In this study, changes of serum IL-4 concentrations at different times corresponded to the difference of the animals’ health status after HP-PRRSV challenge (Table 1, Figures 2 and 3). These findings suggested that at the beginning of the HP-PRRSV challenge, IL-4 down-regulates the inflammatory response and promotes PRRSV replication (Figure 4, Table 2). This may have increased the pathogenicity of PRRSV by down-regulating cellular immunity and thus reduces the ability of the attenuated vaccine to protect the pigs against PRRSV infection. However, later after the HP-PRRSV challenge, high serum concentrations of IL-4 are needed for the development of the adaptive immunity against PRRSV infection, enhancement of the immune stages and production of antibodies (Figure 4).

Functional studies on IL-10 suggest that it has immunosuppressive properties and can prevent the development of Th1-mediated autoimmune diseases [36]. Some viruses, including PRRSV, can induce IL-10 production to inhibit the host immune response and hamper the process of virus clearance [30,37-39]. In our study, piglets challenged with HP-PRRSV alone developed high levels of IL-10 (Figure 4D), which indicated that IL-10 also inhibits the cell-mediated immunity and keeps the longer duration of viremia in HP-PRRSV infection.

The animals that received control antibodies and CH-1R produced higher levels of anti-GP2/GP5 antibodies than those that received Mab2-5G2 or aAb2 (Figure 5B). However, the time of onset and levels of neutralizing antibodies produced in these animals were different (Table 3) in which the piglets received Mab2-5G2 or aAb2 produced neutralizing antibodies at early (21 DPI) stage with low levels even after the HP-PRRSV challenge. The presence of the low levels of neutralizing antibodies (titer < 7, Table 3) before the HP-PRRSV challenge may be responsible for the antibody-dependent enhancement [40-42] leading to poor health status with pneumonia and thymus atrophy (Table 1, Figure 2, Figure 3).

## Conclusions

When the anti-idiotypic antibodies specific for anti-PRRSV GP5 antibodies are present, the use of attenuated PRRSV vaccine CH-1R against HP-PRRSV infection is not recommended. It can result in the altered production of cytokines, low levels of neutralizing antibodies and the poor health status with pneumonia and thymus atrophy.

## Methods

### PRRSV, vaccine and anti-Id

The PRRSV strain used in this study was HP-PRRSV HuN4 strain (GenBank no. EF635006) [43]. The attenuated PRRSV vaccine, CH-1R, was generated from CH-1a, a North American type strain isolated from China (GenBank no. EU807840) [24]. Monoclonal Abs were Mab2-5G2 specific for anti-PRRSV GP5 antibody and a control monoclonal Ab 7H12 specific for avian hepatitis E virus [23,44]. The aAb2s specific for anti-PRRSVGP5 were purified from the sera of pigs experimentally infected with HP-PRRSV HuN4 strain and normal swine IgGs were prepared from PRRSV negative pigs [45]. Each of the Mabs and swine IgGs was mixed with 10% (w/v) aluminum hydroxide adjuvant (Rehydragel, SEPPIC, France) on a magnetic stirrer (at 180 rpm) overnight and stored at 4°C.

### Animal study

Thirty-six 28-day-old PRRSV-free piglets were obtained from a PRRS free farm and randomly divided into 6 groups (Table 4).

**Table 4:** **Groups of piglets with different injections at different time points**^1^

**Time points**^**2**^	**Groups of piglets received injections**^**1**^					
	**1**	**2**	**3**	**4**	**5**	**6**
0	Mab2-5G2	aAb2s	7H12	normal swine IgG	PBS	PBS
14^3^	CH-1R	CH-1R	CH-1R	CH-1R	PBS	PBS
28	CH-1R	CH-1R	CH-1R	CH-1R	PBS	PBS
42^4^	HP-PRRSV	HP-PRRSV	HP-PRRSV	HP-PRRSV	HP-PRRSV	PBS

^1^The amount of IgG each piglet received was 100 μg/kg of body weight and PBS was 2 mls via intramuscularly (i.m.) injection.

^2^The numbers were the days post injection (DPI). 42 DPI was also the 0 days post challenge (DPC).

^3^On 14 DPI, the piglets received CH-1R, the attenuated PRRSV vaccine and generated from CH-1a, a North American type strain isolated from China (GenBank no. EU807840) [24] i.m. on the left side of the neck at 2 doses/piglet diluted in 2 ml of PBS.

^4^On 42 DPI, the piglets were challenged with HP-PRRSV HuN4 strain at 3 × 10^5^ TCID_50_/piglet and also considered as 0 days post challenge (DPC).

The animals were kept in 6 separate rooms, fed with the commercial diets and water ad libitum throughout the experiment. Sera were collected on 0, 7, 14, 21, 28, 35 DPI and 0, 3, 5, 7, 10, 14, 21 DPC and used for the detection of viremia levels of cytokines and antibodies against PRRSV N, GP2/GP5 antigens. The animal experiments were reviewed and approved by Animal Care Committee of Northwest A&F University. At each time point of necropsy (described below) and at the end of experiment, all animals were humanely euthanized.

### Clinical evaluation

Piglets were monitored daily for clinical signs prior to feeding, including anorexia, lethargy, fever, and emaciation. The respiratory scores were given from 0 to 4 (0 - normal; 1 - tachypnea when stressed; 2 - tachypnea at rest; 3 - tachypnea and dyspnea at rest; 4 - severe tachypnea and dyspnea with labored and jerky breathing) [28].

### Necropsy

Three piglets from each Group were euthanized on 14 DPC and the remaining three piglets from each group were euthanized on 28 DPC. At necropsy, the macroscopic lesions of the thymus and lungs were recorded, including the piglets died from PRRS during the experiment.

### Histological examination

Sections of the thymus and lungs were prepared at necropsy on different DPCs and fixed in 10% neutrally-buffered formalin and processed for histological examination using hematoxylin and eosin staining as described previously [46].

### Fluorescent quantitative RT-PCR

TaqMan fluorescent quantitative RT-PCR (qRT-PCR) was performed as described previously [28] to detect PRRSV in sera.

### Detection of serum cytokines

Levels of serum IFN-γ, IL-2, IL-4 and IL-10 were determined using the commercial ELISA kits (Market Inc, USA). The serum concentrations of cytokines (pg/ml) were calculated according to the recombinant porcine cytokines standards supplied in the kits.

### Serology

Serum antibodies against PRRSV N and GP2/GP5 antigens were detected using the HerdCheck® ELISA kit (IDEXX Laboratories, Westbrook, Maine, U.S.A.) and LIVTESTSUISPRRS A/S® kit (Laboratorios Hipra, Amer, Girona, Spain), respectively. The S/P ratio of HerdCheck® ELISA kit greater than 0.4 and a relative index (expressed as a percentage, IRPC) of LIVTESTSUISPRRS A/S® kit greater than 20 were considered positive.

Virus neutralization assays (VN) was performed as described previously [47] to detect the neutralizing antibodies with the following modifications. Briefly, a 2-fold serial diluted serum samples were mixed with an equal volume of HP-PRRSV HuN4 strain solution with a titer of 100 TCID_50_/mL and incubated for 1 h at 37°C. The mixture was transferred to a 96-well plate containing Marc-145 cells. Cytopathic effect (CPE) was recorded for 7 days. The titers of neutralizing antibodies were defined as the reciprocal of the highest dilution that inhibited CPE in 50% of the inoculated wells.

### Statistical analysis

The numerical data were expressed as mean ± S.D. Respiratory scores, virus quantity and serum concentrations of cytokines were analyzed using GraphPad Prism software (version 5.02 for Windows; GraphPad Software Inc.) for variance (ANOVA). A *p* value less than 0.05 was considered statistically significant.

## Abbreviations

PRRS: Porcine reproductive and respiratory syndrome; PRRSV: Porcine reproductive and respiratory syndrome virus; aAb2s: Auto-anti-idiotypic antibodies; i.m.: Intramuscularly; b.w.: Body weight; DPI: Day post-injection; DPC: Days post-challenge.

## Competing interests

The authors declare that they have no competing interests.

## Authors’ contribution

YY and GW performed the experiment, arranged the data for statistical analysis and drafted the manuscript. NK, YGL, YHX, CZ, YM, SQX, QZ and CBW participated in animal studies. JAH, GPZ and XHC analyzed the data and revised the manuscript.EMZ designed the study, analyzed the data and revised the manuscript. All of the authors read and approved the final manuscript.
